# Impact of Homegrown School Feeding Program on Smallholders’ Farmer Household Food Security in Northeastern Nigeria

**DOI:** 10.3390/foods12122408

**Published:** 2023-06-18

**Authors:** Bulus Barnabas, Sylvester Amoako Agyemang, Edwin Zhllima, Miroslava Bavorova

**Affiliations:** 1 Department of Economics and Development, Faculty of Tropical AgriScience, Czech University of Life Sciences Prague, 165 00 Prague, Czech Republic; barnabas@ftz.czu.cz (B.B.); amoako_agyemang@ftz.czu.cz (S.A.A.); 2 Department of Economics and Rural Development Policies, Faculty of Economics and Agribusiness, Agriculture University of Tirana, 1000 Tirana, Albania; edvinzh@ubt.edu.al

**Keywords:** smallholder farmers, food security, school feeding, Nigeria, propensity score matching

## Abstract

In various countries of the global south, school feeding programs are implemented in order to alleviate short-time hunger in children, improved their nutrition and provide employment for food vendors. The impact of these programs is important not only in terms of pupils’ nutrition but also in improving farmers’ livelihoods productivity and food security. This study analyzes the impact of the school feeding program on smallholder farmers’ household food security based on data collected in 2021 from 240 farmers surveyed in northeast Nigeria. Differently from other studies, several econometric methods are used to analyze the data, namely, binary probit regression, propensity score matching, inverse probability weighted adjusted regression, and endogenous switching regression. The results show that about 40% of the beneficial smallholder farmers are food secure compared to 20% of the nonbeneficiary households. Findings revealed that the Homegrown school feeding program (HGSF) positively improved smallholder farmers household food security status across all the models. Results provide evidence for the need to expand school feeding programs vis-a-vis interventions in facilitating farmers’ access to capital and capacity building for better integration in the supply chain.

## 1. Introduction 

School feeding programs (SFPs) serve as an important safety net program to ensure that every child has access to quality education, health, and nutrition. In recent years, the goals of SFPs have been expanded, and some programs have included local smallholder farmers as food suppliers to improve their livelihoods [1]. Such expanded programs are called Homegrown School Feeding Programs (HGSF). HGSF objectives and tools vary from country to country, but they are all characterized by the integration of local food suppliers into the supply chains that provide agricultural commodities or food to schools [2].

Several countries such as Brazil, Ethiopia, Mali, Ghana, India, and South Africa have incorporated direct purchase (decentralized model) of food products from local farmers into their SFP in order to improve the smallholder farmer household food security [3]. The decentralized purchase model of HGSF enables district authorities, firms, schools, and caterers to buy food products directly from smallholder farmers or their organizations [4,5,6]. To facilitate direct purchases and reduce transaction costs, besides the role of the service supplier or the catering company, in some cases, the role of procurement committees is witnessed, which are established and made up of parents’ associations, community members, and teachers [4,5]. Caterers are also supported with information and guidelines for adapting food supply sources to pupils’ nutritional needs [5]. In a decentralized procurement system, caterers are not restricted or guided in their procurement rules, thus giving impetus to purchases from local smallholders [7].

In Nigeria, HGSF is launched in 2016 in order to increase enrollment, attendance, and performance and improve pupils’ nutrition in schools [2,8]. The program is currently benefiting about 9.9 million pupils in 33 states. There are about 107,000 cooks employed who are benefiting, and around 150,000 smallholder farmers are linked to the program, which provides locally sourced ingredients in a decentralized model of input purchase [5,9].

Theoretically, the HGSF instruments have been recognized as having a positive impact on agricultural and overall rural livelihood [10]. Several studies have revealed evidence that HGSF has improved smallholder farmers’ income, livelihood and food security status by increasing the demand for vegetable products, cereals, and other staple foods cultivated by the farmers [11,12,13,14,15]. An increase in opportunities to sell has reduced farmers’ losses encountered, especially vegetable farmers, and improved their profits [16]. The market guarantee through HGSF can stimulate an increase in agricultural productivity and reduce marketing risks for farmers [10,11,17] since they can produce and market nonstaple perishable foods such as vegetables and legumes [18]. Furthermore, the activation of HGSF offers additional benefits of job creation and empowerment to caterers and processors, providing a steady market opportunity for smallholder farmers and thus improving their livelihoods and local economy through the decentralized system of procurement [2,5].

Despite the theoretical and empirical justifications of HGSFs, the potential to improve agricultural productivity and food security among smallholder farmers [10,11,13] is constrained by some conditions related to the sustainability of the HGSF. The first condition is that farmers must be willing to supply food products consistently throughout the school year; the second condition is that the seasonality in local food production can limit continual procurements [4]. For instance, in Malawi, cases of poor linkages between smallholder farmers and school feeding programs have been reported, affecting their effectiveness and collaboration among numerous stakeholders, including government organizations, schools, farmers’ organizations, and caterers [19]. Furthermore, smallholder farmers in Nigeria and other sub-Saharan countries are often dependent on rain-fed agriculture and face seasonal crop production variations. This can cause fluctuations in agricultural produce availability, making it difficult to consistently supply caterers or schools with the required quantity and variety of food items throughout the year [4]. Similarly, many smallholder farmers in Nigeria and other sub-Saharan countries cultivate in remote or rural areas with limited access to infrastructure like roads, storage facilities, and processing plants. This can create difficulties when transporting agricultural produce, ensuring its quality and safety, and keeping it fresh during delivery to caterers [20,21,22].

Considering these limitations, the potential of HGSF to improve farmers’ household food security is a key issue to be explored and the influence of institutional and farm characteristics is to be further analyzed. This study investigates the factors affecting smallholders’ food security with a focus on farmers’ links to caterers under the HGSF. Another way the study investigates the life insecurity of farming households concentrating on the benefits farmers drive by selling their produce to caterers through HGSF which acts as a conduit to a reliable marketplace. Access to HGSF is also observed vis a vis with other factors namely access to credit, input subsidies, market information, and smallholder household characteristics as well as other vectors of food security.

Notwithstanding the large coverage and the relatively long duration, to the best of our knowledge, no prior studies have concentrated on how HGSF instruments affect the food security of smallholder farmers’ households. Furthermore, previous studies conducted across the globe [11,14,23,24] scarcely use factual and counterfactual analyses (experimental versus control). To close the empirical gaps, our study adopts the Propensity Score Matching (PSM), Inverse Probability Weighted Adjusted Regression (IPWRA), and Endogenous Switching Regression (ESR) approaches to control for endogeneity resulting from observed and unobserved individual characteristics [25,26,27,28].

Further research is needed to assess the economic viability of linking smallholder farmers to school feeding programs as it relates to their household food security status. By addressing these research gaps, policymakers, researchers, and practitioners can gain valuable insights and develop evidence-based strategies to effectively link smallholder farmers to school feeding programs, thereby promoting food security, nutrition, and sustainable agricultural practices.

To fill this void and provide useful knowledge to researchers and policymakers, the study answers the following research questions.

- What are the factors affecting smallholder farmer household food security status?
- Does linking smallholder farmers to HGSF improve their household food security?

The study analyzes the impact of the HGSF on smallholders’ food security, based on data collected in February 2021 from 240 farmers surveyed in northeast Nigeria which is a suitable research environment for exploring the benefits of school feeding programs in an area dominated by smallholders, suffering from high food insecurity [5,29] and under continual security risk from Boko Haram attacks [30]. Due to the vicinity of the conflict areas. This sample, being divided into two groups (beneficiary and nonbeneficiary smallholder farmers) with similar characteristics, creates a suitable laboratory for observing the impact of participation on farmers’ performance and food security. As such, this study brings additional findings in the case of a country suffering from food insecurity and life insecurity.

The remainder of this paper is as follows. Section 2 introduces the theoretical background and the review of the literature. The later section presents the methodological approach. Results are presented in Section 4, which is followed by the discussions. In the final section, the main conclusions and implications are provided.

## 2. Theoretical Background and Literature Review

The HGSF is underpinned by the theory of change [31], which is a framework that explains how strategies, activities, or programs contribute to a set of specific outcomes through a series of intermediate outcomes in a systematic way. For instance, [32] used the theory of change as well as the combined group model building (GMB) approach to analyze the HGSF in the Caribbean. An additional theory is a Polytheoretical Model for Food and Garden-based Education in School Settings (PMFGBE), which is a framework of components representing the underpinning forces for adopting the program [33]. On the demand side, the theoretical base is the [34] Theory of Planned Behavior (TPB), which is used to explain changes in individuals’ dietary habits and behaviors that result from individuals’ intentions, which can be influenced by a dietary-induced program.

**Figure 1:**
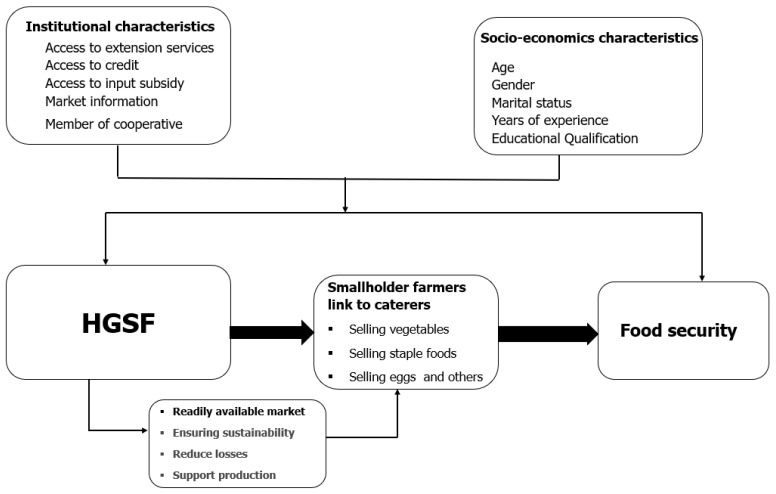
Conceptual framework. Source: Authors illustration, (2022).

Even though there is no unified model of HGSF, the programs aim to tackle both social protection and agricultural development benefits [10]. The literature mainly views the effect of HGSF as an option for providing smallholders a reliable market by increasing farmers’ access to capital to improve production, linking farmers to caterers [35] and food processors to sell their surpluses or during periods when schools are on the break [10,35,36]. These interventions create market opportunities for farmers, reduce their variable costs, and enable them to better utilization of their labor endowment, thus bringing higher incomes, fewer food losses, and greater food security (Figure 1).

For instance, several studies emphasize the effect of linking smallholder farmers with caterers [11,12,13] and food processors [37,38,39,40,41] on the farmer’s household food security status. The effect is mainly indicated by the effect of access on production and income increases [38,42,43] in food security.

In addition to ensuring market space, HGSF has also benefited farmers with access to credit or loans. Access to capital, as is the case of African countries reported [44,45,46] has raised farmers’ revenues.

School feeding programs provide an important new opportunity to assist low-income families and feed hungry children while reserving food at home for others and improving household food security status. Several studies have reported that households, where children benefit from the feeding program, are more likely to be food secure because the food they receive at school supplements the little food they receive at home [17,47,48]. Based on our review of the literature, theoretical framework, and potential explanation research question, we developed a hypothesis.

**Hypothesis** **1.** *Linking smallholder farmers to HGSF improves their household food security.*

*Other controlling variables influence household food security statuses, namely* demographic and institutional variables, such as age, gender of the household head, access to extension service, and input subsidy [42,45,49]. For instance, studies conducted in Nigeria, Pakistan, and Tanzania have demonstrated that an increase in the age of smallholder farmers increases their household food security status [40,49,50]. Contrary to these studies, other authors [51,52] revealed that as age increases, household food security decreases.

There is contrasting evidence in relation to the gender of the household head. Studies by [45,53] reported that female-headed households are better off in terms of food than their male counterparts, while other studies provide evidence for the contrary [42,44,49,50].

The household head’s marital status is also important [42,50], and household size is an important factor in the food security status of smallholder farmers. Thus, larger household size increases the likely chance of the farming household being food-secured [44,45,50]. Several studies, on the other hand, have found that a larger household size among smallholder farmers affects household food security status positively, as demonstrated by [42,49,54].

Farming experience is also a noted vector for food security. Farmers with longer farming experience have a better chance of being food secure than farmers with fewer years of farming experience [42,45].

Education is expected to have a significant impact on food security status. An increase in farmers’ years of formal education by household heads is likely to be food secure [40,42,45,55,56], while the contrary is true for a few authors (see [49]).

As emphasized earlier, institutional characteristics of the environment where farmers operate are important. Several studies analyzed the role of extension services in influencing household food security. Smallholder farmers with access to extension services are more likely to be food secure than their counterparts who lack access to the services [37,44,45,50,55,56].

According to [57], access to market information is the primary driver of market participation. When a household has access to market information, they can make informed decisions about what to produce, when to sell it, whom to sell it to, and what price to accept. They are therefore more likely to have higher cash incomes, which they can use to purchase a variety of foods, including their favorites. In fact, compared to their counterparts, households with access to market information are more likely to be food secure [57,58].

Access to social capital and networks is alluring too. Belonging to one form of cooperative society has improved smallholder farmers’ food security status. Membership affiliation in farmers’ cooperative societies improved smallholder farmer food security status [54,59].

The factors identified are estimated in the case of Northern Nigeria using several analytical models. Differently from other authors such as [11,12,13], we consider the effect of HGSF vis a vis other factors. In addition, use counterfactual analyses in order to estimate the effects on food security.

## 3. Methodology

### 3.1. The Study Area

Nigeria is a country with a rapid increase in population (growth rate of 3.2%) achieving 213 million in 2021. Considering this demographic trend, the country is expected to have 410 million inhabitants by 2050. The mortality rate of below five years is 101 of 1000 live births [60], and food security is a major issue for development.

The study area was chosen due to high levels of household insecurity and acute malnutrition among children, with many at risk of death [61]. This is caused by climate change vulnerability and the Boko Haram insurgency attacks in northeast Nigeria (i.e., our study area). Approximately, 60% of the 13 million out-of-school children in Nigeria live in the Northeast region [29]. More than 800,000 children in Northeast Nigeria are projected to be acutely malnourished by 2021 Food consumption in Northeast Nigeria has worsened compared to previous years, with poor and borderline food consumption (reported by 44% of households) nearly as high as at the peak of the crisis [5,62]. In 2019, an estimated 7.1 million people who lived in the region needed assistance; this included 2 million displaced people from the conflict [63].

#### Definition of the Study Sample

The Homegrown School Feeding Program (HGSF) is a value chain arrangement that encourages local smallholder farmers to grow or produce locally and sell to caterers (food vendors) responsible for feeding pupils in schools. This allows farmers to sell to a readily available market with fewer losses. In our sample, beneficiary smallholder farmers (i.e., treatment variable) are those registered farmers under the program who have been linked to caterers and already selling any of the following to the caterers in the past 1–2 years (i.e., vegetables, staple foods, and egg sales). While our nonbeneficiary smallholders were newly registered farmers under the program, they are yet to be linked with prospective caterers to start enjoying the benefits of the program.

### 3.2. Sampling Procedure and Sample Size

**Figure 2:**
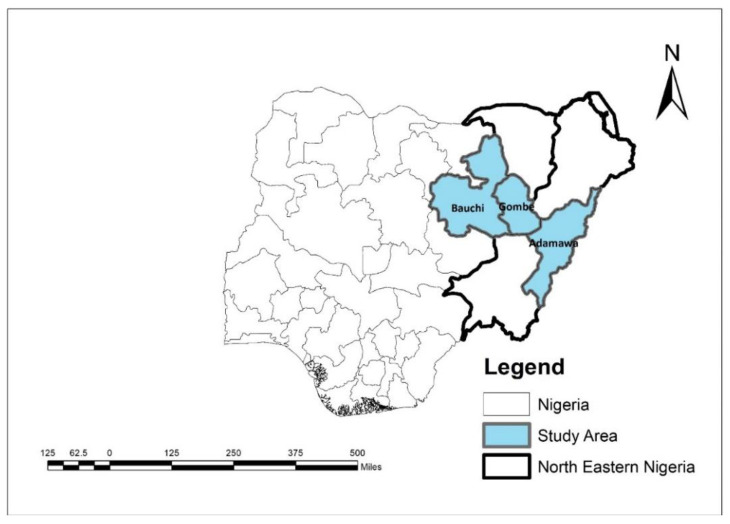
The study area (Author, 2021).

Respondents for the study are HGSF-registered and nonregistered smallholder farmers from across the study area. A multistage sampling procedure was used to select smallholder farmers. The first approach involves the purposeful selection (due to accessibility and low threat of death) of three northeastern Nigerian states, namely, Adamawa, Bauchi, and Gombe; the reason is due to the threat to life due to the Boko Haram attacks and kidnapping. In addition, these areas are from the same agroclimatic zone cultivating the same types of crops and rearing livestock. Figure 2 illustrates the targeted areas.

Stage two involved the selection of four local government areas at random (lottery) from each of the three states, for a total of 12 local government areas (Table 1). In stage three, five wards are drawn at random (lottery) from the initial list of local government areas, yielding a total of 60 wards (a ward: a city or borough administrative division that elects and represents a councilor). In the fourth stage, we used systematic random sampling to select farmers from the program’s registered participants in each ward. Each ward has between 6 and 12 registered farmers, depending on the population size. A registered list of smallholder farmers registered with the program was obtained from the Ministry of Agriculture in their respective states for selection and contact with farmers. Systematic random sampling was used to select 2 farmers from the registered, whereas in 6 wards, 3 registered farmers were selected due to the proportion of registered farmers. According to the program’s objectives, registered farmers will benefit from credit access to support the production, farmers linked to caterers, and farmers linked to processors in situations of excess production.

### 3.3. Data Collection

The study is based on structured face-to-face survey data collected using a mobile phone application “kobotoolbox “. Data was collected from 240 smallholder farmers in three states of Northern Nigeria. The lead author of the paper carried out the interviews.

The study questionnaire, based on a literature review and in-depth interviews, was explicitly designed for smallholder farmers and was divided into four sections. The first section of the questionnaire contains information on the socioeconomic variables of the farmers such as (age, years of farming experience, level of education, marital status, household size, and number of pupils benefiting from the school feeding program). The second section contains information on the benefits of farmers’ involvement in HGSF, such as what type of products farmers sell to caterers. The third section contains information on institutional factors that affect the food security status of smallholder farmers, such as access to credit, access to extension services, access to market information, membership in the cooperative society, access to input subsidies, etc. The fourth section of the questionnaire deals with the measurement of food security using the Food Consumption Score (FCS) indicator, a seven-day recall of food consumed by the household.

The questionnaire was pretested and fine-tuned with 24 smallholders or 10% of the study sample size as recommended [64]. The aim was to assess if the questionnaire components were long enough, and questions were easily understood. The data collected was cleaned using Excel but was coded and analyzed using Stata 14 statistical software.

### 3.4. Data Analysis

#### 3.4.1. Probit Model

A probit model was used to determine factors affecting smallholder farmer household food security status. Average marginal effects were estimated and are presented in the results section, as demonstrated by [39,40,41,45].

The probit model in the following form was used:

(1) $$Y_{ik}=\beta_{1}X_{i}+\varepsilon_{i}$$

where *X_i_* represents a set of all explanatory variables presented in the study, *β*_1_ is a vector of estimated parameters, and *ε_i_* is an error term. *Y_ik_* is the level of consumption score where 0 = poor and borderline food security with FCS up to 35; 1 = acceptable food security with FCS higher than 35 points.

The system of equations describing the binary choices of smallholder farmers is given as follows:

(2) $$\begin{array}{r} Y_{ik}=1\;ifY_{ik}>0\\ 0\;otherwise \end{array}$$

#### 3.4.2. Empirical Strategy

##### Propensity Score Matching and Endogenous Switching Regression

Due to observable and unobservable bias, determining the causal effects of HGSF on potential outcome indicators (household food security status) is not straightforward. Controlling for both observable and unobservable characteristics through the random assignment of individuals to treatments is necessary for accurate impact measurement. Selection bias may persist in the absence of random assignment because observed and unobserved characteristics of individuals may influence the likelihood of receiving treatments as well as outcome indicators. To account for endogeneity bias, we use propensity score matching (PSM), inverse probability weighted adjusted regression (IPWRA), and endogenous switching regression (ESR) techniques in this study [25,65,66,67]. These analytical frameworks used help to eliminate selection bias (i.e., observable and unobservable) associated with establishing conditional causality with observational data when randomized trials are infeasible [46,68,69]. To determine the average difference in the outcome variable between treated and untreated households, PSM first matches each treated household to a comparable untreated household. In other words, we want to know: “*What would have happened to the food security status of a smallholder farmer who benefited from HGSF (treated) if that same farmer did not benefit from the HGSF (control)?*”. The Average Treatment Effect (ATT) is described by [70] as:

ATT = *E*[*Y*(1) − *Y*(0)|*T* = 1] (3)

where *Y*(1) and *Y*(0) are outcome indicators (in this case, household food security status). T is the treatment indicator. However, in our dataset, we only see *E*[*Y*(1)|*T* = 1] and *E*[*Y*(0)|*T* = 1] is missing. In essence, we cannot observe the household food security status of treated households if they had not been treated [25,65]. A simple comparison of household food security status of those with and without treatment status introduces self-selection bias into the estimated impacts. The extent of self-selection bias is formally reported.

*E*[*Y*(1) − *Y*(0)|*T* = 1] = *ATT* + *E*[*Y*(0)|*T* = 1 − *Y*(0)|*T* = 0] (4)

PSM reduces the bias introduced by observables by constructing comparable counterfactual households for treated households. PSM assumes that there are no systematic differences in unobservable characteristics between treated and untreated households once households are matched with observables [71]. Given this conditional independence assumption and the overlap requirements, the ATT is calculated as follows:

*ATT* = *E*[*Y*(1)|*T* = 1, *p*(*x*)] − *E*[*Y*(0)|*T* = 0, *p*(*x*)] (5)

However, in the presence of misspecification in the propensity score model, ATT from PSM can still produce biased results [26,66]. The use of inverse probability-weighted adjusted regression (IPWRA) could be a remedy for such misspecification bias. According to [27] IPWRA estimates will be consistent in the presence of treatment/outcome model misspecification, but not both. As a result, the IPWRA estimator has the double-robust property, which ensures reliable estimates by accounting for misspecification in both the outcome and the treatment model [28,46]. Ref. [70] proposed two steps for estimating ATT in the IPWRA model. Assume the outcome model is represented by a linear regression function of the form Y_i_ = α_i_ + φ_i_x_i_ + ɛ_i_ for I = [0 1] and the propensity scores are given by *p*(*x*; $\overset{⁀}{\gamma }$). The propensity scores are estimated in the first step as *p*(*x*; γ). In the second step, we use linear regression to estimate (α_0,_ φ_0_) and (α_1,_ φ_1_) using inverse probability weighted least squares as the regression model.

(6) $$\frac{min}{\alpha_{0},\varphi_{0}}\sum_{i}^{N}(Y_{i}-\alpha_{0}-\varphi_{0}x_{i})/p\left(x,\overset{⁀}{\gamma }\right)\;if\;T_{i}=0$$

(7) $$\frac{min}{\alpha_{1},\varphi_{1}}\sum_{i}^{N}(Y_{i}-\alpha_{1}-\varphi_{1}x_{i})/p\left(x,\overset{⁀}{\gamma }\right)\;if\;T_{i}=1$$

The ATT is then computed as the difference between Equations (6) and (7)

(8) $$\mathrm{ATT}=\frac{1}{N_{w}}\sum_{i}^{N_{w}}\left[\left({\overset{⁀}{\alpha }}_{1}-{\overset{⁀}{\alpha }}_{0}\right)-\left({\overset{⁀}{\varphi }}_{1}-\varphi_{0}\right)x_{i}\right]$$

where, (${\overset{⁀}{\alpha }}_{1}$,${\overset{⁀}{\varphi }}_{1}$) are estimated inverse probability-weighted parameters for HGSF beneficiary households while (${\overset{⁀}{\alpha }}_{0}$,${\overset{⁀}{\varphi }}_{0}$) are estimated inverse probability-weighted parameters for nonbeneficiary households. Finally, *N_W_* stands for the total number of treated households.

Matching techniques can only overcome selection bias caused by observables, regardless of misspecification bias adjustments. When unobservable heterogeneity, such as a farmer’s inherent skill, causes endogeneity, estimates of the matching technique will be biased. As a result, we used the endogenous switching regression (ESR) model in the final step to account for both observed and unobserved bias [72,73]. The ESR method solves the endogeneity problem by estimating the selection and outcome equations with full information maximum likelihood (FIML) [28,74].

Furthermore, proper ESR identification necessitates the use of at least one instrumental variable that influences the treatment rather than the outcome of interest. The possible instrument in the first ESR model, for example, “farmers benefiting HGSF”, was identified as “access to input subsidy”. Thus, from the question “Do you have access to input subsidy?”, we created a dummy variable “those with access to input subsidy” that takes a value of 1 and 0 otherwise. The assumption is that farmers who have access to input subsidies have a better chance of benefiting from HGSF. However, access to input subsidies are not supposed to have a direct impact on the outcome variable of interest because simply having access to input subsidies does not directly improve or decrease household food security, as adopted [75].

We assume that a particular farming household would consider receiving treatment, i.e., benefit from the HGSF if the expected benefit of the treatment (in terms of food security status) is positive. Let *F*_0_ be the food security status of farmer households not benefiting the HGSF (i.e., control group), and let *F*_1_ be the corresponding food security status of the treatment group. The farmer will choose to be in the treatment of the food security improvement defined as, *Y*_i_^*^ = F_1_ − F_0_, which is positive. However, the food security status improvement that the farmer derives from treatment (*Y*_i_^*^) is a latent variable determined by observed characteristics (*Z*_i_) as follows:

(9) $${Y_{i}}^{*}=\beta^{0}+\gamma Z_{i}+\mu_{i}\;\mathrm{with}\;T_{i}=\left\{\frac{1if{Y_{i}}^{*}>0}{0if{Y_{i}}^{*}\leq 0}\right.$$

Variables affecting expected gains from having benefited the HGSF are represented by the vector Z. The conditional outcome function can then be specified as an ESR model in the following way.

Regime1: Y_1__i_ = γ_1 × 1i_ + ᵋ_1i_
*if* T_i_ = 1 (10)

Regime2: Y_2__i_ = γ_2_ × _2i_ + ᵋ_2i_
*if* T_i_ = 0 (11)

where Y_1i_ is the outcome indicator for treated farmer households and Y_2i_ is the outcome indicator for untreated farmer households, and x_i_ is a vector of exogenous variables. The outcome variable’s error term is in the selection equation (i.e., Equation (9)) and the outcome equation (i.e., Equations (10) and (11) the error terms are assumed to have a trivariate normal distribution with a mean of zero and a covariance matrix (Ω) in the following way:

$$\Omega =\left[\begin{array}{ccc} o_{u}^{2} & o_{1µ} & o_{2µ}\\ o_{1µ} & o_{1}^{2} & .\\ o_{2µ} & . & o_{2}^{2} \end{array}\right]$$

where $o_{u}^{2}$ = *var*(µ_i_), $o_{1}^{2}$ = var(ᵋ_1_), $o_{2}^{2}$ = (ᵋ_2_), $o_{1µ}$ = *cov*(µ_i_, ε_1_), $o_{2µ}$ = *cov*(µ_i_, ε_2_). Furthermore, $o_{u}^{2}$ = is estimable up to a scale factor and can be assumed to be equal to 1 [67] and cov(ε_1_, ε_2_) is not defined as Y_1_ and Y_2_ cannot be observed simultaneously. Moreover, the correlation between the error term of the selection equation and the outcome equation is not zero (i.e., corr(µ_1_, ε_1_) ≠ 0 and corr(µ_1_, ε_2_) ≠ 0), which creates selection bias. ESR addresses this selection bias by estimating the inverse mills ratios (λ_1__i_ and λ_2__i_) and the covariance terms ($o_{1µ}$ and $o_{2µ}$) and including them as auxiliary regressors in Equations (10) and (11). If $o_{1µ}$ and $o_{2µ}$ are significant, we reject the absence of selection bias. In addition, $o_{1µ}$ < 0 represents positive selection bias (i.e., households with above-average food security are more likely to choose to be in the treatment). The ESR model estimates can then be used to estimate ATT (Average treatment effect on untreated households) as follows:

(12) $$E(Y_{1i}|T_{i}=1)=\gamma_{1}x_{1i}+\lambda_{1i}\;o_{1µ}$$

(13) $$E(Y_{2i}|T_{i}=0)=\gamma_{2}x_{2i}+\lambda_{2i}\;o_{2µ}$$

(14) $$E(Y_{2i}|T_{i}=1)=\gamma_{2}x_{1i}+\lambda_{1i}\;o_{2µ}$$

(15) $$E(Y_{1i}|T_{i}=0)=\gamma_{1}x_{2i}+\lambda_{2i}\;o_{1µ}$$

Equations (12) and (13) along the diagonal of Table 2 represent the actual expectations observed in the sample. Equations (14) and (15) describe the counterfactual expected outcome (15). In addition, we calculate the average treatment of the treated “ beneficiaries’ farmers” on the treated (ATT) as the difference between Equations (12) and (14) following the [76],

(16) $$\mathrm{ATT}=E(Y_{1i}|T_{i}=1)-E(Y_{2i}|T_{i}=1)=x_{1i}(\gamma_{1}\;-\gamma_{2})+(o_{1µ}-\;o_{2µ})\lambda_{1i}$$

which represents the impact of HGSF on the household food security status of a beneficiary smallholder farmer. For the impact on the household food security status of nonbeneficiary smallholder farmers, we calculate the effect of treatment (HGSF) on the untreated (ATU) as the difference between Equations (13) and (15).

(17) $$\mathrm{ATU}=E(Y_{1i}|T_{i}=0)-E(Y_{2i}|T_{i}=0)=x_{2i}(\gamma_{1}\;-\;\gamma_{2})+(o_{1µ}\;-\;o_{2µ})\lambda_{2i}$$

To account for the effects of heterogeneity, we adapt for beneficiaries of HGSF. For example, beneficiary farmers may have a higher household food security status than nonbeneficiaries, even though they benefit due to unobservable characteristics such as their skills. We chose to adapt because of the difference between (a) and (d) (see Table 2).

(18) $${\mathrm{BH}}_{1}\;=\;E(Y_{1i}|T_{i}=1)-E(Y_{1i}|T_{i}=0)=(x_{1i}\;-\;x_{2i})\;\lambda_{1i}+o_{1µ}(\lambda_{1i}-\lambda_{2i})$$

The difference between Equations (16) and (17) is “transitional heterogeneity,” or whether the effect of benefiting from HGSF is larger or smaller among beneficiaries or nonbeneficiaries in the counterfactual case that they did benefit (i.e., ATT and ATU).

(19) $${\mathrm{BH}}_{2}\;=E(Y_{2i}|T_{i}=1)-E(Y_{2i}|T_{i}=0)=(x_{1i}-x_{2i})\;\lambda_{2i}+o_{2µ}(\lambda_{1i}-\lambda_{2i})$$

T_i_ = 1 if farmers are beneficiaries; Ai = 0 if farmers are nonbeneficiaries.

Y_1i_: changes in household food security status if farmers are beneficiaries.

Y_2i_: changes in household food security status if farmers are nonbeneficiaries.

ATT: Average effect of the treatment (i.e., beneficiaries) on the treated (i.e., beneficiaries’ farmers of HGSF).

ATU: the effect of the treatment (i.e., HGSF) on the untreated (i.e., nonbeneficiaries’ farmers of HGSF).

BH_1_: the effect of base heterogeneity for beneficiaries’ farmers (i = 1), and nonbeneficiaries’ farmers (i = 2)

TH = (ATT − ATU), i.e., transitional heterogeneity

### 3.5. Sample Description

#### 3.5.1. Selection of Variables in the Models

Several studies indicate that demographic, socioeconomic, and institutional factors can all affect smallholder farmers’ household food security status. The following proxy variables that may affect smallholder farmers’ household food security were identified in the previous empirical literature as detailed in Section 2. These include age, education level, marital status, years of farming experience, extension service, input subsidies, and market information, as well as participation in HGSF and types of food items farmers sell to caterers.

#### 3.5.2. Description of Variables in the Probit Model

##### The Food Consumption Score

The World Food Programme developed the FCS as a frequency-weighted dietary diversity score [77]. The FCS is the sum of the number of times a food group from the household dietary score was eaten in the previous seven-day period. Information on the frequency of consumption in the week prior of cereals, tubers, pulses, vegetables, fruits, meats and fish, milk, sugar, and oil, multiplied by the weight (importance in the diet) assigned to each group by the World Food Program [78]. The scores are then classified into three categories: poor (<21.5), borderline (21.5–35), and acceptable (>35) categories. The model used is as follows:

*FCS* = *a*_1_*b*_1_ + *a*_2_*b*_2_ + *a*_3_*b*_3_…………*a*_8_*b*_8_ (20)

where a = weight of each food, 1–8 = Food group, and b = Frequency of food consumption (days consumed from each food group over the previous 7 days).

Table 3 furthermore, displays the variables the main variables, as identified from the literature, which are expected to influence the food security of smallholder farmers as proxied by the food consumption score. A majority (67.1%) of the respondents were male with a mean age of 42.09. Slightly more than 88% of the respondents are married. The result indicated that 35% of the smallholder farmers obtained a secondary education and about 31% of farmers had no formal education. Respondents’ average length of farming experience is 17.67 years. The results, furthermore, revealed that among the 126 beneficiary farmers majority (55.6%) of the farmers supply different forms of vegetables to caterers’ access, while 27.8% of the registered farmers supply staple food and 16.7% of them supply eggs and others to caterers involved in cooking for the pupils. funding under the school feeding program for farmers to produce. Approximately 18% of the farmers had access to extension service delivery. In addition, 35.0% of the farmers had access to input subsidy, and 42.5% had access to market information. Slightly more than 22.5% of the respondents were members of a cooperative group.

## 4. Result

### 4.1. Sociodemographic Information of Smallholder Farmers

Table 4 compares sociodemographic information from beneficiaries and nonbeneficiary smallholder farmers. The findings reveal that HGSF beneficiary smallholder farmers have a mean age of 41.98 years, while nonbeneficiary smallholder farmers have a mean age of 42.20 years, indicating a nonsignificant difference in the age of the farmers. There is no statistically significant difference in the gender of beneficiary farmers—no significant dominance of male farmers in one group, although the number of male farmers is higher for the nonbeneficiary group (i.e., 69%) than the beneficiaries (65%). The beneficiary household size has a mean of 7.7 people, while the nonbeneficiary household size is 8.2 people, implying a nonsignificant dominance in household size. There is no statistically significant difference in years of farming experience of HGSF beneficiary smallholder farmers and nonbeneficiary smallholder farmers with the former having a mean of 17.38 years of farming experience, while the latter has a mean of 17.98 years of farming experience.

Findings revealed that there is a significant difference in educational attainment between HGSF beneficiaries with a mean of 3.23 and nonbeneficiary farmers with a mean of 2.40. About 75% of the beneficiary HGSF smallholder farmers had access to credit, whereas 12% of nonbeneficiary farmers had access to credit. Access to input subsidies revealed that (18%) of HGSF beneficiaries of smallholder farmers have access to input subsidies, whereas (30%) of the nonbeneficiary farmers had access to input subsidies. Implying a significant difference in their access to input subsidy, indicating that nonbeneficiary farmers have more access to input subsidy. We assume that due to the significant difference in their educational attainment, access to credit, and access to input subsidies, they are likely to influence the outcome variable in the study (i.e., household food security status). We therefore adopted the PSM/ESR model to help us deal with any observed bias that may arise due to statistically significant differences between beneficiary and nonbeneficiary smallholder farmers in sample selection process.

### 4.2. Household Food Security Status of Farmers

Table 5 result shows the food security status of smallholder farming households’ status. Findings revealed that 0.5% of beneficiary farming households fell within the poor category against 9.26% of the nonbeneficiary, 60.32% of the beneficiary households were in the borderline category, while 70.56% of the nonbeneficiary fell within the borderline and 39.18% of the HGSF beneficiary were within acceptable levels compared to 20.18% of the nonbeneficiary households. Inferring that most of the households were food insecure. This is consistent with the World Bank Group’s report [79], which reported that up to 73% of households in northeast Nigeria are poor. Similarly, the National Bureau of Statistics [60] stated that about approximately 78% of the population in northern Nigeria live below the country’s poverty level.

### 4.3. Factors Affecting Smallholder Farmers’ Household Food Security Status

The results of the probit model presented in Table 6 indicate that participation in the HGSF has a statistically positive and significant effect on the household food security status of smallholder farmers, with a marginal effect of 0.404. The result implies that smallholder farmers who participate in the HGSF are likely to experience a 40% increase in their household food security status. Findings revealed that increase in age of smallholder farmer negatively affect his household food security status with a marginal effect of −0.008, implying a year increase is likely to negatively affect the household food security by 0.8%. Smallholder farmers access to credit has a statistically positive and significant effect on household food security status with a marginal effect of 0.270, meaning that access to credit is likely to increase household food security by 27%. Farmer contact with an extension agent has a statistically significant positive relationship with smallholder household food security, with a marginal effect of 0.061. This means that farmers who have contact with an extension agent are expected to have 6% higher food consumption than farmers who do not have access to extension services.

### 4.4. Effect of Homegrown School Feeding Program on the Food Security Status

The result of treatment effect estimates on HGSF smallholders on their household food security using alternative estimation techniques are presented in Table 7 below. Columns 1, 2, and 3 present treatment effect results based on PSM, IPWRA, and ESR specifications. The results are robust across all estimation strategies, demonstrating the impact of HGSF on smallholder farmer household food security status. The results from all the three models show that the HGSF program has a positive effect on the food security status of smallholders, though the impact is heterogenous in respect to the approach. From Table 7, estimate of the PSM method (model 1) shows that farmers benefiting from the HGSF would have been 4.9 points worse off if they had not benefited from the program. When using the IPWRA specifications (model 2), the household food security status of smallholder farmers increases by 3.3 points for benefiting from HGSF. The ESR model (model 3), where we accounted for both observable and unobservable bias, indicates that a beneficiary smallholder farmer’s household food security status increases by 5.6 points more than if that same farmer had not benefited from the program (see Appendix A). The estimation produces different results because PSM produces bias estimates in the presence of misspecification in the model and the IPWRA remedies such misspecification bias using the double-robust property, which ensures reliable estimation by accounting for misspecification both in the outcome and treatment model.

## 5. Discussion

The result was triangulated in our study using four different models to assess the impact of HGSF on smallholder farmers’ household food security using alternative estimation techniques: Probit regression (dummy variable beneficiary and nonbeneficiary), PSM, IPWRA, and ESR specifications. The results are robust to all estimation strategies and show a positive impact of HGSF on the food security status of smallholder households.

The results imply that the HGSF has created an avenue for linking smallholder farmers with caterers to create a readily available market (value chain) for farmers to sell their produce with limited losses due to perishability and fast return which in turn helps to improve their household food security status. This is in line with the findings of other authors [11,14,23,24,80], who found that farmers who collaborated with caterers to sell their goods saw an improvement in their household food security status. This implies that when farmers are linked to selling their produce to caterers, it creates a reliable market and reduces postharvest losses usually encountered by smallholder farmers. Farmers’ linkage to caterers tends also to increase these farmers’ household incomes and expenditures, improving their food security status. Several authors [39,40] reported that farmers with market links have a reliable market and are more commercialized, with significantly higher producer prices and household food security status than those without such linkages.

Furthermore, farmers selling vegetables to caterers under the HGSF have been shown to improve smallholder house food security status. When smallholder farmers have a market guarantee, they are more likely to produce and market nonstaple perishable foods such as vegetables and legumes [18,81]. Similarly, farmers selling eggs to caterers has demonstrated to increase in their household food security status. The HGSF has improved smallholder farmers’ income, livelihood, and food security status by increasing the demand for vegetable products, cereals, and other staple foods cultivated by the farmers [12,13]. For example, farmers in Indonesia reported having more opportunities to sell their products as a result of the HGSF’s purchases [16].

Thus, our hypothesis that stated linking smallholder farmers to HGSF improves household food security was approved. The study findings allow us to recommend that the program be expanded to nonbeneficiary areas in the studied study site in order to extend the program’s positive effects on smallholder farmers’ household food security status.

The result of treatment effect estimates reveals that access to credit has been shown to improve the food security of smallholder farmer households. Making credit available to all participating farmers will thus provide them with funds to purchase needed farming incentives, resulting in improved smallholder household food security. This finding supports [82,83], who reported that smallholder farmers with access to credit can provide a variety of options for improving agricultural production, including access to inputs that can boost productivity and household food security. Furthermore, access to the extension has ensured higher food security. In support of this finding, several authors [45,55,56] reported that access to extension service delivery improves smallholder farmers’ household food security status.

## 6. Conclusions and Implications

The research examined how HGSF impacted smallholder farmer households’ food security in Northeastern Nigeria. Overall, HGSF has a positive impact on smallholder farmers’ household food security status. The influence of HGSF, namely, farmers selling vegetables to caterers and farmers selling eggs, positively influences household food security. The implication of our findings is that a consistent and reliable market has been created for farmers to sell their products, thereby increasing their household income and food security. Thus, the better synergy between farmers and caterers will strengthen the supply chain relevant added value, which will provide a reliable market for the farmer to sell her or his product, thereby improving household food security. This research on linking smallholder farmers to school feeding programs can advance the scientific understanding of various interconnected domains, including food security, nutrition, agriculture, economics, logistics, social dynamics, and policy developments.

The limitations of the study are the missing baseline data on farmers’ previous food security status and lack of other food security indicators such as household income food security indicators. Thus, to make better policy recommendations, it is critical to emphasize the need for a follow-up longitudinal study that considers the program’s long-term viability and potential long-term impacts. To obtain more robust and reliable information, baseline data should be included in future studies. Baseline data may assist in better understanding the farmers’ households’ food security status in the areas before different programs are implemented in the future. As a support, a system of monitoring and supervision should be put in place to ensure the HGSF program’s success. This intervention would have also practical benefits, since it will help to increase the percentage of smallholder farmers involved in the program as well.

## Figures and Tables

**Table 1:** Sample Selection.

State	LGAs	Wards	Beneficiary Farmers	Nonbeneficiary Farmers
Adamawa	Yola north	5	11	10
	Demsa	5	10	9
	Numan	5	11	10
	Mayo-Belwa	5	10	9
Bauchi	Alkaleri	5	10	9
	Bauchi	5	11	10
	Dass	5	10	9
	Katagum	5	11	10
Gombe	Akko	5	11	10
	Billiri	5	10	9
	Gombe	5	11	10
	Bajoga	5	10	9
Total	12	60	126	114

LGA—Local governmental area.

**Table 2:** Conditional Expectations, Treatment, and Heterogeneity Effects.

Subsamples	Decision Stage		Treatment Effects
	Beneficiaries	Nonbeneficiaries	
Beneficiaries’ farmers	(a) E(Y_1__i_|T_i_ = 1)	(c) E(Y_2__i_|T_i_ = 1)	ATT
Nonbeneficiaries’ farmers	(d) E(Y_1__i_|T_i_ = 0)	(b) E(Y_2__i_|T_i_ = 0)	ATU
Heterogeneity effects	BH_1_	BH_2_	TH

Note: (a) and (b) represent observed expected farmers’ benefiting the HGSF; (c) and (d) represent counterfactual expected farmers’ not benefiting the HGSF.

**Table 3:** Description of variables imported into the models (*n* = 240).

Variables	Description and Measurement	Mean	Std. Dev.
Dependent Variable			
Food security indicators			
Food consumption score	0 = poor and borderline (up to 35), 1 = acceptable (>35)	0.30	0.46
Independent Variables			
Household head characteristics			
Age	Age of household head (years)	42.09	8.48
Gender	Male = 1, Female = 0	0.67	0.47
Marital status	Married = 1, unmarried = 0	0.89	0.31
Years of experience	Farming experience in years	17.67	8.91
Educational Qualification	Quranic Edu. = 1, primary = 2, secondary = 3, NCE = 4, graduate = 5, postgraduate = 6	2.83	1.44
Household characteristics			
Household size	The household size in numbers	7.94	3.88
Households with children benefiting from SFP	Yes = 1 No = 0	0.61	0.49
Homegrown school feeding program			
HGSF program	Beneficiary farmers = 1 Nonbeneficiary = 0	0.53	0.50
Institutional variables			
Access to extension services	Yes = 1 No = 0	0.18	0.38
Access to credit	Yes = 1 No = 0	0.45	0.50
Access to input subsidy	Yes = 1 No = 0	0.24	0.42
Market information	Yes = 1 No = 0	0.03	0.16
Member of cooperative	Yes = 1 No = 0	0.21	0.14

NCE: National Certificate of Education, HGSF: Home-Grown School Feeding Program, SFP: School Feeding Program.

**Table 4:** Sociodemographic and institutional between the beneficiary and nonbeneficiary farmers.

Variables	Beneficiary Farmers (*n* = 126)	Nonbeneficiary Farmers (*n* = 114)	Mean Difference	*t*-Statistics
	Mean ± S.D.	Mean ± SD		
Age of farmers	41.98 (8.77)	42.20 (8.19)	−0.22	0.20
Gender	0.65 (0.48)	0.69 (0.46)	−0.04	0.69
Marital status	0.86 (0.35)	0.93 (0.35)	−0.07	1.81
Household size	7.71 (3.82)	8.19 (3.95)	−0.48	0.95
Years of farming experience	17.38 (9.03)	17.98 (8.80)	−0.60	0.52
Educational Qualification	3.23 (1.50)	2.40 (1.23)	0.83 ***	4.69
HH Children benefiting SFP	0.56 (0.50)	0.66 (0.48)	−0.10	1.496
Access to credit	0.75 (0.43)	0.12 (0.32)	0.63 ***	12.616
Access to extension services	0.21 (0.41)	0.14 (0.36)	0.07	1.153
Access to input subsidy	0.18 (0.38)	0.30 (0.46)	−0.12 **	2.242
Market information	0.02 (0.15)	0.03 (0.16)	−0.01	0.123
Cooperative membership	0.02 (0.15)	0.02 (0.13)	0.00	0.338
FCS (Household)	36.88 (11.55)	29.64 (7.56)	7.24 ***	5.682

Source: Own survey 2021, *** 1% level of significance; ** 5% level of significance.

**Table 5:** Food Security Status of the Farming Household.

FCS	Profile	Beneficiary Farmers % (*n* = 126)	Nonbeneficiary Farmers% (*n* = 114)
0–21	Poor	0.5	9.26
21.5–35	Borderline	60.32	70.56
>35	Acceptable	39.18	20.18

FCS: Food Consumption Score.

**Table 6:** Factors affecting the level of food security—results of binary probit model.

Variable	Marginal Effect	Std. Err.
Social safety net program		
HGSF status	0.404 ***	0.087
Household head characteristics		
Age	−0.008 *	0.004
Gender	0.002	0.044
Marital status	−0.016	0.065
Years of farming experience	0.003	0.004
Educational Qualification	0.022	0.019
Household characteristic		
Household size	0.010	0.007
Households with children benefiting SFP	0.022	0.043
Institutional characteristic		
Access to credit	0.270 ***	0.087
Extension service delivery	0.063 *	0.065
Input subsidy	0.101	0.066
Market information	0.289	0.338
Number of observations	240	
Constant	4.348	
LR chi^2^	52.56	
Pseudo R^2^	0.251	
Prob > chi2	0.000	

Statistical significance: * = 10% level, *** = 1% level.

**Table 7:** Effect of HGSF on smallholder farmer household food security status.

Variables	Average Treatment Effect on the Treated (ATT)		
	PSM	IPWRA	ESR
	1	2	3
HGSF	4.931 **	3.258 **	5.554 ***
	(1.997)	(1.582)	(0.476)
N	240	240	240

Robust standard errors are reported in parentheses, level of significance; 0.01 = ***; 0.05 = **. Source: Authors’ estimations.

**Figure A1:**
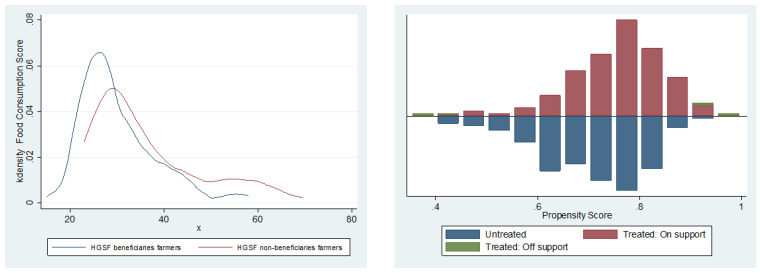
Kdensity estimate and propensity scores distribution between HGSF beneficiaries and nonbeneficiaries smallholder farmers on their household food security status. Source Authors’ own elaboration.

## Data Availability

The data used to support the findings of this study can be made available by the corresponding author upon request.

## Appendix A

**Table A1:** Endogenous switching regression results in the effect of HGSF on the household food security status.

			Effect of HGSF on Household Food Security			
	HGSF Status		HGSF Beneficiaries		Nonbeneficiaries	
Variables	Coef.	Std. Err.	Coef.	Std. Err.	Coef.	Std. Err.
Age	0.022	0.022	−0.386	0.196 **	−0.156	0.218
Gender	−0.116	0.211	2.811	2.173	−1.128	2.003
Household size	0.015	0.037	0.893	0.302 ***	−0.591	0.389
Years of experience	−0.015	0.022	−0.085	0.191	0.210	0.220
Education qualification	0.619	0.079 ***				
Access to input subsidy	−0.771	0.268 ***				
Market information	0.688	0.418 *				
Constant	−3.127	0.852 ***	41.064	6.132 ***	45.647	5.997 ***
/lns1	2.275	0.082 ***				
/lns2	2.354	0.062 ***				
/r1	−0.695	0.223 ***				
/r2	0.032	0.266				
sigma_1	9.726	0.805				
sigma_2	10.531	0.651				
rho_1	−0.601	0.142				
rho_2	0.032	0.265				
Log-likelihood	−1000.408					
Wald test χ^2^ (4)	4.67					
LR test of independent equations χ 2 (1) 8.64 ***						

*** 1% level of significance; ** 5% level of significance; * 10% level of significance.

**Table A2:** Average expected effect of HGSF on smallholder farmer household food security status; treatment and heterogeneity effects.

	Decision Stage		
Sub-Samples	HGSF Beneficiaries	Nonbeneficiaries	Treatment Effect
HGSF beneficiaries’ farmers	39.853 (0.344)	34.299 (0.319)	TT = 5.554 *** (0.476)
Nonbeneficiaries’ farmers	32.706 (0.340)	31.741 (0.292)	TU = 0.965 *** (0.964)
Heterogeneity effects	BH_2_ = 7.147	BH_1_ = 2.558	TH = 4.589 ***

BH^i^: the effect of base heterogeneity for HGSF beneficiaries (i = 1) and nonbeneficiaries of HGSF (i = 0). *** 1% level of significance.
